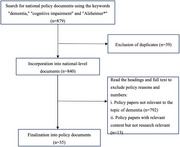# Evolution of Strategic Framework of Policies Related to Dementia Care and Prevention in China from 2000 to 2024

**DOI:** 10.1002/alz70860_101778

**Published:** 2025-12-23

**Authors:** Yuting Luo, Qian Xiong, Xin Guan, Huali Wang

**Affiliations:** ^1^ Nanchang University, Nanchang, Jiangxi, China; ^2^ School of Public Policy and Administration, Nanchang University, Nanchang, Jiangxi, China; ^3^ Institute of China's Rural Revitalization Research, Nanchang, Jiangxi, China; ^4^ Department of Social Work and Social Administration, The University of Hong Kong, Hong Kong, Hong Kong SAR, China; ^5^ Dementia Care and Research Center, Peking University Institute of Mental Health (Sixth Hospital), Beijing, China; ^6^ NHC Key Laboratory for Mental Disorders, Beijing, Beijing, China; ^7^ National Clinical Research Center for Mental Disorders, Beijing, China

## Abstract

**Background:**

Dementia has emerged as a critical public health issue on the global policy agenda. Over the past 25 years, China has increasingly recognized the significant challenges posed by dementia and has made substantial strides in formulating relevant policies. This study aims to analyze the strategic framework of China's dementia policy development to provide valuable insights for other low‐ and middle‐income countries as they draft their national action plans.

**Method:**

Policy documents were systematically searched and retrieved from the official websites of pertinent policy agencies. The evolutionary phases of dementia policies were identified based on the timeline of policy issuance. A comparative thematic analysis was conducted to explore the evolution of policy content. Additionally, WordCloud analysis was utilized to examine the frequency of relevant terms within these documents. Collinear network relationship diagrams were then created to illustrate the evolving trends in the relationships among key terms and activities related to dementia care.

**Results:**

A total of 35 policy documents were included in this study (see flow chart in Figure 1). Three distinct phases were identified over the past 25 years: an incipient phase (2002–2014), a development phase (2015–2018), and a boom phase (2019–2024). Themes within dementia policies have progressively expanded and enriched across these phases. There has been a notable shift in focus from “intervention” during the incipient phase to “dementia prevention” in the boom phase, with themes such as “screening and evaluation” and “public education” gaining prominence. Stakeholder involvement has diversified to include entities like “community,” “institutions,” and “social workers.” Moreover, the scope of potential beneficiaries has broadened from “patients” to encompass “family members” and “caregivers.” The number of nodes related to dementia policies has increased, and their interconnections have strengthened over time.

**Conclusion:**

Over the past 25 years, the themes, content, and stakeholders involved in China's dementia‐related policies have expanded significantly. Furthermore, the interconnection among key terms and content has grown stronger. These findings offer valuable references for advancing national dementia initiatives and updating dementia action plans in low‐ and middle‐income countries.

Funding: Jiangxi Province Key Research Base Program for Philosophy and Social Sciences (23ZXSKJD28)

Figure 1. Flow chart